# Vitrification of isolated mice blastomeres using a closed loading device

**DOI:** 10.1186/1477-7827-7-17

**Published:** 2009-02-19

**Authors:** Amr Kader, Ashok Agarwal, Rakesh Sharma, Tommaso Falcone

**Affiliations:** 1 Center for Reproductive Medicine, Glickman Urological and Kidney Institute and Obstetrics & Gynecology and Women's Health Institute, Cleveland Clinic, Cleveland, Ohio, USA; 2 Center of Surgical Innovation, Technology and Education, Cleveland Clinic, Cleveland, Ohio, USA; 3 Department of Obstetrics and Gynecology, Alexandria University, Alexandria, Egypt

## Abstract

Isolated blastomeres obtained by embryo biopsy serve mainly for preimplantation genetic screening. Blastomeres are undifferentiated embryonic cells that include all the embryo genetic information. A lot of developing technologies may benefit by the efficient cryopreservation of blastomeres for future potential use, especially for stem cell culture and differentiation control. We are hereby reporting for the first time the feasibility of preserving individual isolated blastomeres in microvolumes in a closed vitrification system. Using a cryotip and propagation in microvolumes, isolated mice blastomeres were vitrified and warmed with 100% post-warming survival.

## Background

Blastomeres can be isolated from cleavage stage embryos by embryo biopsying. This technique is used for preimplantation genetic diagnosis (PGD) of inherited diseases, or pre-implantation genetic screening (PGS) for aneuploidy. The most commonly applied method for diagnosis is fluorescent in situ hybridization (FISH). In this method, 7 to 10 chromosomes are checked routinely for aneuploidy and trisomies and in some cases up to 15 chromosomes [1]. The complete genomic hybridization assay (CGH) enables more accurate and reliable analysis of entire genetic material using a single blastomere. [2]. However, the clinical benefit of diagnosis of aneuploidy using a single blastomere is still controversial. Embryo biopsy is now recommended to be limited to genetic diagnosis of inherited disease (PGD) rather than aneuploidy screening which recently proved not to improve the clinical outcome in randomized control trials.[3,4]

Other potential uses of isolated blastomeres are:

1) Serve as a bank of genetic information.

2) Future development of stem cells. Successful in vitro culture was achieved with embryonic and extra-embryonic stem cells developing from single mouse blastomeres.[5,6] Research is directed towards the differentiation of these pluripotent cells towards specific cell lines from human single blastomeres. This is of immense value with great improvements anticipated in the diagnostic and therapeutic techniques relying on stem cells. [7-10] Recently, the development of human embryonic stem cell lines has been made possible using a single blastomere of a 4 cell stage embryo.[11,12] and later stage embryos.[13]

3) Development of isolated blastomeres *in vitro *has been proposed as a possible marker for the development of the embryo they originated from.[14] and

4) It is also known that the transfer of trophectodrm cells together with the embryo transfer increases the chances of successful pregnancy due to its production of polypeptide products that help maintain an early pregnancy. [15]. This procedure has been used in animal husbandry, with either fresh or vitrified trophectoderm cells [16]. Isolated blastomeres should therefore be considered as a potential self source of extraembryonic tocopherodermic cells that may be used as an adjuvant to embryo transfer.

A reliable method of cryopreservation of isolated blastomeres would therefore seem mandatory for the pursuit of any of these previously mentioned purposes. The cryopreservation of blastomeres is already an established technique used in pisciculture. This is traditionally done with slow freezing. A recent single report described its successful vitrification in microdrops [17]

Vitrification is the ultra-rapid method of cryopreservation. It avoids intracellular ice crystallization that can damage the cells in slow freezing through achieving extremely high cooling rate that wouldn't allow ice crystals to form. Vitrification can be achieved by direct or indirect contact with liquid nitrogen. Vitrification has been successfully used in cryopreserving oocytes, embryos and blastocysts. [18-21] With the increasing concerns about liquid nitrogen contamination, closed loading systems that can achieve adequate cooling and warming rates are investigated.[22] Despite the increasing applications of closed vitrification devices, there has been no description in the literature as of now on the use of a closed vitrification system to vitrify isolated blastomeres.

In this technical brief, we describe for the first time the vitrification and warming techniques of isolated individual mice blastomeres using the cryotip as a closed vitrification loading device.

## Methods

### Mouse embryo biopsy

Cryopreserved commercially available 8 cell mouse embryos (Embryotech Laboratories, Inc., Wilmington, MA) were thawed and incubated for 4 hours. After incubation, only grade (A) embryos (clear, equal cells) with no fragmentation were selected for biopsy. Embryo biopsy was performed with the aid of two hydraulic micromanipulators (Narishige, Tokyo, Japan) and microsyringes (IM-6; Narishige, Tokyo, Japan) mounted on an inverted microscope (Nikon, Eclipe TE200, Tokyo, Japan). The embryos were immobilized with the holding micropipette. Assisted hatching was done using Irvine scientific acidified tyrode's solution (Santa Ana, CA). One or 2 blastomeres were removed through the created zonal opening using an embryo biopsy needle. Twelve individual blastomeres were obtained from 8 different embryos for further vitrification.

### Vitrification of blastomeres

Vitrification and warming were done using Irvine scientific vitrification media (Irvine, Santa Ana, CA) and cryotip loading device according to the manufacturer's recommended protocol with slight modification.[23] The blastomeres to be vitrified were first placed in 10 μl drop of the equilibration solution (7.5% of each DMSO and EG and 20% serum substitute supplement SSS, (Irvine, Santa Ana, CA) for 3 minutes then transferred into a series of 3 droplets of 10 μl of the vitrification solution (with 15% of each DMSO and EG glycol and 20% serum substitute supplement) for 5 seconds in the 1^st ^and 2^nd ^droplet and for 60 seconds in the last droplet. The blastomeres were loaded in the cryotip loading device with 1 μl of the vitrification solution. Two blastomeres were loaded in one cryotip, 2 other cryotips were loaded with 3 blastomeres in each. A fourth cryotip was loaded with 4 blastomeres. Loaded cryotips were sealed from both sides with heat pulse sealer and immediately transferred to liquid nitrogen.

Warming was carried out after at least 24 hours by immediately placing a cryotip in a 37°C water bath for 3 seconds. In a Petri dish, 1 droplet of 10 μl of thawing media, 2 droplets of 10 μl of dilution media (0.5 M sucrose, 20% SSS). The warmed cryotip was then cut at its ends and the contents were brought down near to the thawing solution droplet (1 M sucrose, 20% SSS). The blastomeres retrieved after warming were placed in thawing solution for 1 minute, each of the dilution solutions for 2 minutes. While the blastomeres were in the 2^nd ^droplet of dilution solution, 3 droplets of 10 μl of washing media (20% SSS) were placed in the same Petri dish. The blastomeres were finally transferred in the washing solution droplets for 3 minutes in each droplet.

### DNA damage

To detect cell death in blastomeres, we used the deoxynucleotidyl transferase (TdT) – mediated dUTP nick-end labeling (TUNEL) (in-situ cell death detection system; Roche Diagnostic Corporation, IN, USA). After warming, blastomeres were fixed in 4% paraformaldehyde in PBS (pH 7.4) for 1 hour at room temperature then stained for DNA damage as previously described. [23] After fixation, blastomeres were washed in PBS containing 0.3% polyvinylpyrrolidone (PBS/PVP) and permeabilized in 0.5% Triton X-100 on ice for 2 min. The blastomeres were then washed in PBS/PVP and incubated in (TUNEL) reaction cocktail at 37°C for 1 hour in the dark. Blastomeres were washed in PBS and mounted in DAPI (4',6-diamidino-2-phenylindole) containing Vectasheild antibleaching solution (Vector Labs, Burlingame, CA, USA). The mounting medium drops were covered with cover slides The slides were examined with a Leica TCS-SP2 laser scanning spectral confocal microscope (Leica Microsystems, Heidelberg, GmbH) using an HCX Plan Apo 63X, 1.32 NA, oil immersion objective at zoom 2. The specimen was excited at 364 nm (UV) for DAPI and 488 nm for fluorescein isothiocynate-conjugated-TUNEL. TUNEL positive cells emit a green fluorescence, while DAPI emits blue fluorescence from all blastomeres. Thus, TUNEL negative blastomeres will only show blue fluorescence. The emitted fluorescence from each of the two labels was detected with two separate photomultiplier detectors whose spectrophotometer slits were set for 400–490, 500–550 respectively.

Both positive and negative controls were included for validation of the DNA staining technique and DNA damage detection. Positive controls were produced by exposing intact 8 cell stage embryos to 1 U DNAse I (Sigma Aldrich, St. Louis, MI) for 15 minutes at room temperature. Different embryos that were not exposed to DNAse I or TUNEL reaction cocktail were used as negative controls. Control embryos (positive and negative) as well as isolated blastomeres were checked for any green (TUNEL positive) signals to detect any DNA damage or apoptosis after the vitrification and warming. [23]

## Results

**Figure 1:**
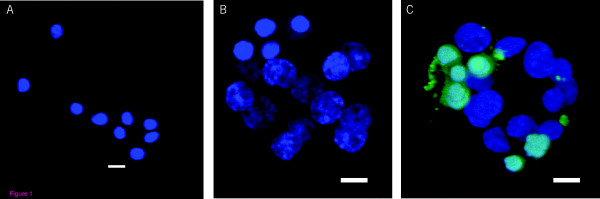
**Vitrified-warmed blastomeres examined for DNA damage by TUNEL staining (A): Isolated blastomeres showing no TUNEL signals; (B): negative control biopsied embryos with no TUNEL signals; (C): positive control biopsied embryos showing TUNEL (Green) signals**.

None of the vitrified-warmed blastomeres showed any positive TUNEL signals (Fig. 1A), compared to the negative (Fig. 1B) and positive (Fig 1C) controls.

## Discussion

The objective of this experiment was to test the feasibility of vitrification of isolated blastomeres in a closed vitrification system.

Kang et al have previously reported the possibility of vitrifying isolated mice blastomeres derived from 2 cell mouse embryos in 0.25 ml plastic straws [24]. The in vitro development rate of vitrified-thawed blastomeres was similar to fresh control. In our report, we cryopreserved blastomeres derived from 8 cell mouse embryos after embryo biopsy similar to the technique used for PGD.

Slow cryopreservation and vitrification to a higher extent have shown the possibility to induce apoptosis in the blastocyst's blastomeres. However, this effect is expected to be related to special characters of the blastocyst: size, high number of cells as well as the presence of the blastocele.[23] Therefore, it is more likely that the individual blastomere would better tolerate cryopreservation in general.

The 8 cell embryo blastomere is produced in the course of embryo biopsying for PGD. The embryo biopsy at 8 cell is still compatible with further embryo development, making the 8 cell blastomere a suitable candidate for future developments in stem cell research. It has been recently demonstrated that human embryonic stem cells can be generated from blastomeres generated by techniques similar to PGD and without destruction of the embryo. [13] This avoids the ethical issues related to the destruction of embryos for research purposes and allows ther investigators to research the potential(s) of generating self embryonic stem cells at 8 cell development stage while allowing ongoing development of this embryo to maturity and delivery.

From a cryopreservation perspective, the difference in size between the two and eight cell stage blastomeres is expected to make the 8 cell stage more tolerant to cryopreservation in general, but more difficult to process for cryopreservation or for retrieval after thawing.

In addition to its efficacy in maintaining the post-warming survival of the blastomeres, the vitrification protocol we applied uses micro-volume solutions. This makes vitrification a more practical option for freezing individual blastomeres compared to slow freezing. It is more convenient to manipulate and retrieve a single or a smaller number of individual blastomeres in a micro-volume environment (10 μl drops, 0.1 μl loading device). Available slow freezing systems would require a much larger volume (~0.25 ml for cryogenic straws) that would make manipulation and retrieval of isolated, small, single blastomeres extremely difficult. It is however to be noted that during vitrification and rapid blastomere dehydration, it becomes difficult to keep track of the blastomeres in the media as they will change their position in the solution with the rapid change of viscosity. Therefore, we have used a even smaller volume of solution (10 μl) to propagate the blastomeres than what is usually recommended (20 μl).

The use of a closed vitrification system avoids the liquid nitrogen contamination risks. Different closed systems have been reported to have rapid enough cooling and warming rates and therefore achieves successful vitrification with post-warming survival that can match open systems.[22,25,26]

To the best of our knowledge, this is the first report of successful vitrification and warming of isolated individual mice blastomeres, derived from 8 cell embryo stage and in a closed loading vitrification device. Additional information on any possible cellular or metabolic effects following 8 cell stage blastomere conventional cryopreservation or vitrification should be further investigated.

The addition of vitrification of isolated blastomeres as a tool in assisted reproduction programs opens new possibilities for other applications such as PGD, genetic banking, stem cell production and any possible future diagnostic or therapeutic use.

## Conclusion

The cryotip can be used for vitrification of isolated blastomeres. Vitrification of isolated blastomeres from PGD biopsy is a simple and reliable method for storing single or a small number of individual blastomeres for possible diagnostic or therapeutic procedures that can be immediately or remotely applied, notably by generation of human embryonic stem cells.

## Competing interests

The authors declare that they have no competing interests.

## Authors' contributions

AK carried out the embryo biopsy, vitrification, TUNEL staining, imaging and worked in drafting the manuscript. AA participated in the design of the study and supervised the analysis. RS helped in the study coordination and drafting the manuscript. TF conceived the study, participated in the design of the study and supervised the analysis. All authors read and approved the final manuscript.
